# Effects of Seat Vibration on Biometric Signals and Postural Stability in a Simulated Autonomous Driving Environment

**DOI:** 10.3390/s25196039

**Published:** 2025-10-01

**Authors:** Emi Yuda, Yutaka Yoshida, Kunio Sato, Hideki Sakamoto, Makoto Takahashi

**Affiliations:** 1 Innovation Center for Semiconductor and Digital Future, Mie University, Tsu 514-0102, Japan; 2 Department of Management Science and Technology, Graduate School of Engineering, Tohoku University, Sendai 980-8577, Japan; makoto.td@tohoku.ac.jp; 3 Faculty of Engineering, Mie University, Tsu 514-0102, Japan; yoshida@sansui.mie-u.ac.jp; 4 Alps Alpine Co., Ltd., Tokyo 145-8501, Japan; kunio.sato@alpsalpine.com (K.S.); hideki.sakamoto@alpsalpine.com (H.S.)

**Keywords:** seat vibration, heart rate variability, postural stability, autonomous driving, vigilance, skin temperature, psychophysiology, aging

## Abstract

This study investigated the physiological effects of seat vibration during prolonged sitting in a simulated autonomous driving environment. Eleven healthy participants (3 young adults and 8 older adults) viewed a 120-min highway driving video under two conditions: rhythmic seat vibration (2 Hz, mimicking natural respiration) and no vibration. Physiological and behavioral metrics—including Psychomotor Vigilance Task (PVT), seat pressure distribution, heart rate variability (HRV), body acceleration, and skin temperature—were assessed across three phases. Results demonstrated that seat vibration significantly enhanced parasympathetic activity, as evidenced by increased HF power and decreased LF/HF ratio (*p* < 0.05), suggesting reduced autonomic stress. Additionally, seated posture remained more stable under vibration, with reduced asymmetry and sway, while the no-vibration condition showed time-dependent postural degradation. Interestingly, skin surface temperature was lower in the vibration condition (*p* < 0.001), indicating a possible thermoregulatory mechanism. In contrast, PVT performance revealed more false starts in the vibration condition, particularly among older adults, suggesting that vibration may not enhance—and could slightly impair—cognitive alertness. These findings suggest that low-frequency seat vibration can support physiological stability and postural control during prolonged sedentary conditions, such as in autonomous vehicles. However, its effects on vigilance appear limited and age-dependent. Overall, rhythmic vibration may contribute to enhancing passenger comfort and reducing fatigue-related risks, particularly in older individuals. Future work should explore adaptive vibration strategies to balance physiological relaxation and cognitive alertness in mobility environments.

## 1. Introduction

In recent years, the development of autonomous driving technology has significantly advanced, promising greater convenience, improved road safety, and reduced driver burden. However, even in highly automated systems, the necessity for human drivers to remain alert and ready to take control in emergency or transitional situations persists. The shift in driver roles—from active vehicle operation to passive monitoring—poses new ergonomic and physiological challenges. Among these, maintaining vigilance and minimizing fatigue during prolonged periods of low engagement is of critical concern. This issue is especially salient for elderly drivers, who may increasingly rely on automated driving functions to maintain mobility and independence into older age.

One potential approach to address this challenge lies in optimizing the design of the driving environment to support natural human rhythms and promote sustained attention [1,2,3,4,5,6,7,8]. Similarly, in the automotive context, it is essential to consider how vehicle interiors, interfaces, and stimuli can be adapted to the evolving needs of drivers.

Currently, many autonomous driving systems impose a paradoxical strain on users by requiring continuous alertness despite offering minimal physical or cognitive engagement. This mismatch can lead to unnatural tension, increased fatigue, and even impaired performance in critical moments. The goal, therefore, should not only be to automate driving functions but also to create an environment that helps drivers maintain optimal physiological and mental states for monitoring and control. For instance, AI-integrated systems could utilize real-time biometric data to assess the driver’s condition and adjust the level of automation accordingly. If signs of fatigue or distraction are detected, the system could temporarily increase automation and offer supportive stimuli to promote arousal and attention.

This study aims to explore one such supportive strategy: the application of rhythmic vibration through the vehicle seat. Specifically, we investigated whether low-frequency vibration (2 Hz), synchronized with the natural respiratory rhythm, could positively influence physiological parameters such as heart rate variability (HRV), body surface temperature, postural stability, and cognitive alertness during prolonged periods of passive driving. In this context, seat vibration was considered not merely as a comfort feature but as a subtle physiological stimulus capable of supporting driver awareness and homeostasis during extended periods of inactivity.

This study reflects a broader vision for aging-friendly vehicle design, where older individuals can retain driving privileges longer by utilizing technology not only to automate but also to assist. In such vehicles, the transition between manual and automatic control would be dynamic, context-sensitive, and guided by real-time assessments of driver capability. The current study contributes to this goal by examining whether simple, non-invasive interventions such as seat vibration can subtly support physiological balance, reduce the rate of postural fatigue, and improve the overall human–machine interaction in autonomous driving contexts. It seeks to bridge the gap between technology and human factors, ensuring that advancements in vehicle automation enhance—not hinder—driver well-being; this work provides a preliminary step toward more adaptive, human-centered autonomous driving systems.

## 2. Materials and Methods

### 2.1. Participants

Total of 11 healthy adults participated in this study, including 3 young male participants (mean 21 ± 1 years old) and 8 older adults (mean 67.6 ± 3.7 years old, 3 females). All participants were free from any major cardiovascular or neurological disorders and were not taking medications that could affect autonomic nervous system function, such as beta-blockers. These conditions were confirmed through verbal screening. To eliminate the influence of substances that may affect physiological responses, participants were instructed to refrain from consuming caffeine or alcohol for at least 12 h prior to the experiment. Participants were recruited from the Silver/Human Resources Center, all participants received a thorough explanation of the study’s purpose, procedures, and potential risks before providing written informed consent. This study was approved by the Ethics Committee and conducted in accordance with the Declaration of Helsinki (Graduate School of Information Sciences, Tohoku University Ethics Committee Approval No. 85, and Approval Date 27 November 2023).

### 2.2. Experimental Protocol

**Figure 1:**
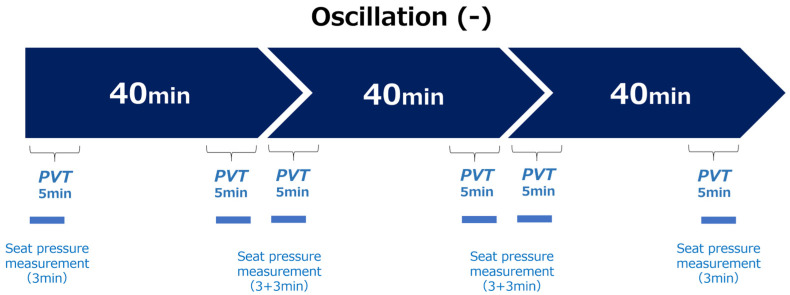

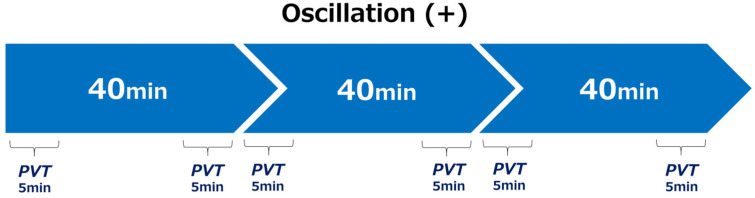
Experimental Protocol.

**Figure 2:**
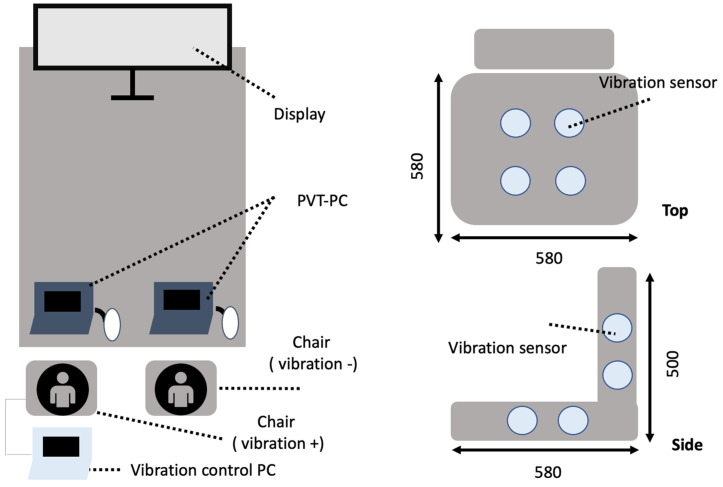
Experimental Layout.

Each participant completed two 120-min sessions, totaling 240 min, while watching a video simulating autonomous driving. The experiment was divided into three 40-min phases, with each phase including the measurement of the PVT and seat pressure mapping at the beginning and end. PVT was conducted for 5 min, and seat pressure was recorded for 3 min per measurement point (Figure 1 and Figure 2).

A pressure distribution sensor system (Azwil, Takano Co., Ltd., Nagano, Japan) was used to measure the seated pressure (see Supplementary Materials). The external dimensions were 600 mm × 600 mm (seat pressure detection area was 397 mm × 397 mm), the number of sensing points was 196 (14 × 14), and the pressure range (F.S.) was 200 mmHg (the detected pressure value was set to 1.2 times F.S.). The measurement accuracy was the average pressure ±10%F.S., and the coefficient of variation was 10%. The communication method was Wi-Fi, and the data was measured by installing P-MAP β version (Azwil’s Windows PC application) on a computer. CSV output was performed via this application. In addition, the sensor was calibrated before the experiment began. The sensor part was made of flexible cloth that does not feel strange when in contact with a person, and the wireless module and battery were integrated into the communication part, so it can be used wirelessly without the power cable getting in the way. Body pressure distribution is detected at sensing points arranged in the detection area, and the detected data is transmitted in real time to a mobile device via Wi-Fi communication.

The electrocardiogram (ECG) RR intervals and body acceleration were recorded using a Holter monitor (Cardy PICO, PICO03; Suzuken Co., Ltd., Nagoya, Japan). The body surface temperature was measured from the left wrist using a skin temperature sensor (Silmee™ Bar type, Silmee™ W22; TDK Corporation, Tokyo, Japan). The sampling frequencies were 125 Hz for ECG RR intervals, 31.25 Hz for body acceleration, and 1 Hz for body surface temperature. When measuring the RR interval, the electrocardiogram electrodes were attached at the NASA lead position. The NASA lead is a measurement method that is excellent for observing P waves because it is difficult to obtain electromyograms due to the potential difference between the upper and lower borders of the sternum. The ECG and acceleration data were exported as CSV files using the Cardy PICO Analyzer software 05 (Suzuken Co., Ltd., Nagoya, Japan). The skin temperature data were exported as CSV files using the software provided by TDK Corporation for use with the Silmee™ W22.

The experiment lasted 120 min, divided into three phases of 40 min each (phases 1–3). In the oscillation (−) condition, a 5-min PVT and seat pressure measurement were performed at the beginning and end of each phase. In the oscillation (+) condition, only the PVT was performed. This is because the influence of vibration makes it impossible to accurately measure seat pressure in the oscillation (+) condition.

(1) Visual Stimulus

To simulate autonomous driving, participants viewed a video showing highway driving from Tokyo Interchange (IC) to Kyoto via the Tomei Expressway (https://youtu.be/SHwIQUbgSIo accessed on 1 September 2025). The video was presented at a constant speed with changing road environments and traffic conditions, displayed on a 27-inch monitor. Participants were seated in a chair resembling a car driver’s seat to replicate the sensation of being in an autonomous vehicle.

(2) Vibration Conditions

Participants sat in a chair designed to mimic a car seat, experiencing one of two conditions:

- Vibration (Oscillation+): A rhythmic vibration at 2 Hz was applied to the seat to simulate the natural rhythm of spontaneous breathing (~4 s per cycle). The 2 Hz frequency was specifically chosen because it provides rhythmic stimulation without inducing discomfort, aligning with the natural breathing cycle and the resonant frequency of the seated human body. Four actuators located from the buttocks to the back were sequentially activated at 0.5-s intervals. (The low-frequency range used in this experiment (approximately 1–20 Hz) aligns with the definitions established in ISO 2631-1 and previous studies on the perceptual and physiological effects of whole-body vibration. In particular, the 2 Hz frequency applied in this study was selected because it closely matches the natural resonant frequency of the seated human body. This frequency has been associated with modulation of arousal and postural control in prior research, making it a relevant parameter for investigating the physiological effects of rhythmic seat vibration.)
- No Vibration (Oscillation−): Participants sat in the same chair without any vibration and watched the same video in a static condition.

The respiratory cycle is a very basic physiological parameter that varies depending on an individual’s state (resting, exercising, sleeping, etc.) and age, but generally follows a 4-s cycle. According to previous studies, the typical respiratory rate for adults at rest is 12–20 breaths per minute, which corresponds to a range of 0.16–0.33 Hz [9,10,11]. The actuators used to generate the seat vibrations were small flat coin-type vibration motors (uxcell DC vibration motor, DC 3V, silver-tone, China). Each motor has a diameter of 10 mm, a thickness of 3 mm, a weight of 6 g, and wire length of 100 mm. These compact disk-shaped motors are commonly used in devices such as smartphone vibration alerts. The vibration is produced by an eccentric rotating mass (ERM) mechanism, in which a small weight is mounted off-center on the motor shaft. When the motor is powered and rotates, the asymmetric mass generates oscillatory forces, thereby converting the electrical signal into mechanical vibration. In this study, four such actuators were embedded along the seat from the buttocks to the back to deliver sequential rhythmic stimulation synchronized to the experimental condition.

(3) Experimental Design

A crossover design was used in which each participant completed both vibration and no-vibration sessions. Half of the participants (N = 6) began with the vibration condition followed by the no-vibration condition, while the remaining participants (N = 5) experienced the conditions in the reverse order. A washout period of at least 15 min was provided between sessions to minimize carryover effects.

The crossover design has the advantage of reducing inter-subject variability and reducing the number of subjects required compared to a parallel group design, because each subject serves as his or her own control group. It is a widely used method in chronic disease research and intervention efficacy evaluation, and a crossover design was adopted for the experimental protocol in this study.

(4) Measured Variables

PVT: Conducted at the start and end of each phase to evaluate attention and alertness. Participants responded to visual stimuli appearing at random intervals, and reaction times were recorded over a 5-min period. VT measures the level of alertness from the speed of reaction by repeatedly pressing a button when a number is displayed. It is used as an index of fatigue and lack of sleep. This reaction time and the number of reaction delays are measured. In addition to reaction time, reaction delays (lapses) and the number of mistakes (false starts, the number of times the button was pressed by mistake before the number was displayed) were also measured. In this experiment, the PVT software (PC-PVT 2.0) was installed on a laptop PC (CF-SV7, 12.1-inch WUXGA (1920 × 1200) size, Panasonic, Tokyo, Japan). Fatigue was indirectly assessed through performance on the Psychomotor Vigilance Task (PVT). Specifically, changes in reaction time over the course of the experiment were analyzed. A progressive increase in reaction time is known to reflect a decline in sustained attention and is considered an indicator of accumulating mental fatigue. Although this method does not provide a direct physiological measurement of fatigue, it is widely accepted as a behavioral marker of reduced vigilance. Thus, the PVT served as a practical tool for evaluating the temporal dynamics of cognitive alertness during prolonged sitting conditions.

Seat Pressure Mapping: Conducted at the same time points as the PVT to assess posture changes. A 16 × 16 pressure sensor matrix was used to continuously record seat pressure distributions during 3-min intervals. In this experiment, the entire experimental protocol of 120 min was divided into three phases of 40 min each. The pressure sensor was installed at the beginning and end of each of the three phases (the experiment was performed six times in total). In this study, the center of pressure (*COP*) was calculated from the seat pressure sensor using the following formula:

(1) $${COP}_{x}=\frac{\sum_{j=1}^{M}\cdot \sum_{j=1}^{N}j\cdot p_{ij}}{\sum_{j=1}^{M}\cdot \sum_{j=1}^{N}p_{ij}}$$

(2) $${COP}_{y}=\frac{\sum_{j=1}^{M}\cdot \sum_{j=1}^{N}i\cdot p_{ij}}{\sum_{j=1}^{M}\cdot \sum_{j=1}^{N}p_{ij}}$$

The formulas show the *COP* in the horizontal direction (*x*-axis) and the *COP* in the vertical direction (*y*-axis) when the coordinate system has the upper left corner of the sensor array as the origin (0, 0) and each pressure value is *p*_*ij*_ (*i* row, *j* column, *i* vertical direction, *j* horizontal direction). *COP* cannot be defined when the denominator, the total pressure, is zero (e.g., when no one is seated).

**Figure 3:**
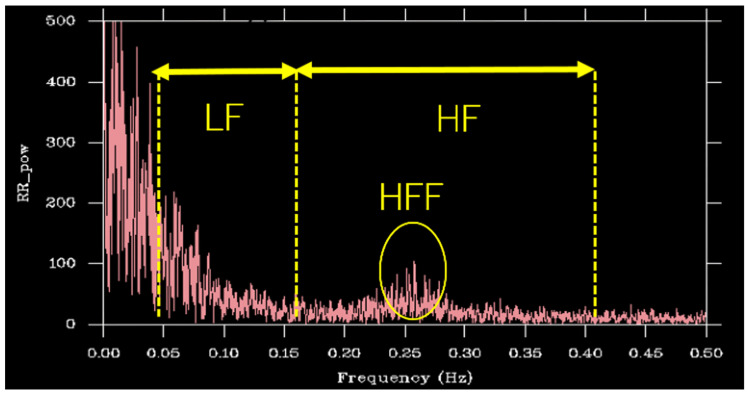
Power spectrum analysis of heart rate variability (one example).

Heart Rate Metrics: Autonomic cardiac activity was evaluated using a Holter ECG recorder (Cardy pico 303, Suzuken Co., Ltd., Nagoya, Japan). From the measured RR intervals, several HRV indices (LF, HF, LF/HF ratio, etc.) were calculated. Power spectral density was estimated from the periodogram using the discrete Fourier transform (Table 1, Figure 3). There’s no specific artifact handling during the vibration phase because our task doesn’t involve closed-loop control like gripping a handle. Such actions typically introduce noise, but its absence here means our data remains clear. Consequently, RRI analysis is feasible without needing additional noise reduction.

Skin Surface Temperature: Peripheral skin temperature was continuously recorded using a wrist-worn wearable sensor (Silmee 22, TDK Corporation, Tokyo, Japan). Temperature data were averaged over 1-min intervals to track changes associated with autonomic regulation and compare thermal responses between conditions.

Participants were instructed to remain in a relaxed state throughout the experiment. All sensors and electrodes were attached before the experiment began, and participants were given at least 10 min to acclimate to the equipment to reduce any discomfort that might affect the measurements.

(5) Postural stability evaluation

The most common and detailed information in postural stability evaluation is the center of pressure (COP), measured using a force plate or a stabilometer. COP excursion is a direct indicator of how stable the body is standing. The total excursion of the COP is the total distance traveled by the COP, and the larger the value, the more unstable the posture. The larger the area of the ellipse or rectangle drawn by the COP’s trajectory, the more unstable the posture. The faster the average speed at which the COP moves, the more unstable the posture. In addition, a bioaccelerometer attached to the chest is suitable for non-invasive and daily evaluation of body sway and activity. The standard deviation (RMS value) of bioacceleration, which indicates the magnitude of body sway, is obtained from the triaxial acceleration data, and the larger it is, the more unstable it is. Postural changes (for example, transition from a sitting position to a standing position) can be confirmed from the triaxial acceleration data of the chest.

The vertical axis is power, and the horizontal axis is frequency. The yellow lines indicate the low frequency (LF) region, high-frequency (HF) region, and HF (respiratory-frequency band). Respiratory rate of 0.25 Hz (i.e., breathing 0.25 times per second) means that the respiratory cycle is 4 s (1/0.25 = 4). This corresponds to a respiratory rate of approximately 15 breaths per minute. This breathing pattern is associated with autonomic nervous system activity, particularly with increased parasympathetic nervous system activity.

### 2.3. Statistical Analysis

To evaluate the effects of vibration, we tested the null hypothesis (H_0_: no effect of vibration) against the alternative hypothesis (H_1_: vibration has an effect). Prior to comparison, the distribution of each dataset was assessed using the Shapiro–Wilk test to confirm normality. Given the crossover design in which each participant experienced both conditions, paired *t*-tests were employed to compare outcomes between the vibration and no-vibration sessions. A significance level of *p* < 0.05 was adopted for all statistical tests, using SPSS software (version 28).

## 3. Results

### 3.1. PVT

**Figure 4:**
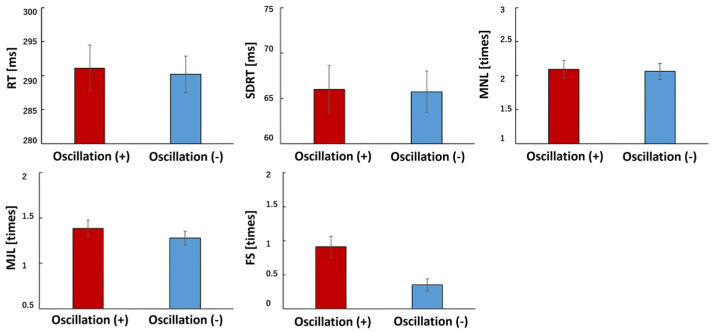
PVT analysis (Oscillate + and Oscillate −). Oscillate (+): N = 66, Oscillate (−): N = 66, using paired *t*-test. The red bar shows the average value of Oscillation (+), and the blue bar shows the average value of Oscillation (−). RT is reaction time, SDRT is standard deviation of reaction time, MNL is minor lapses (500 msec to 800 msec), MJL is major lapses (800 msec), and FS indicates flying start. MNL, MJL, and FS indicate the number of occurrences. RT (*p* = 0.741), SDRT (*p* = 0.931), MNL (*p* = 0.848), MJL (*p* = 0.33), FS (*p* = 0.002).

**Figure 5:**
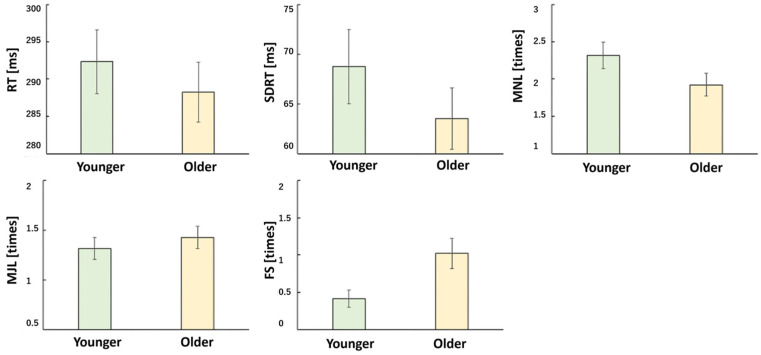
PVT analysis results (young vs. older characteristics). Younger: N = 36, Older: N = 48, independent Samples *t*-test. The green bar shows the average value of Younger, and the yellow bar shows the average value of Older. RT (*p* = 0.493), SDRT (*p* = 0.279), MNL (*p* = 0.1), MJL (*p* = 0.494), FS (*p* = 0.011). Older subjects tend to false start more often.

Participants in the vibration condition (Oscillation+) tended to exhibit more false starts compared to the no-vibration condition (*p* = 0.002). Additionally, older adults showed a higher number of false starts than younger adults (*p* = 0.01) (Figure 4 and Figure 5).

### 3.2. Seat Pressure Distribution

**Figure 6:**
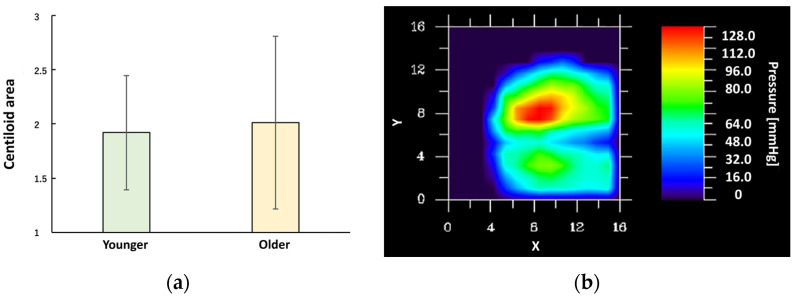
Regional analysis of center of gravity sway caused by seated pressure (young vs. older characteristics). (**a**) Younger: N = 18, Older: N = 48, independent Samples *t*-test (*p* = 0.944). (**b**) shows the distribution of seated pressure as a heat map (an example of visualization software created based on values output from a sensor). The balance of load from left to right and front to back and left to right, as well as the position of the center of gravity, are calculated and displayed. As the experiment progressed to the second half, there was a tendency for the balance to become worse. In (**b**), the *y* axis represents the back and the *x* axis represents the right side. In other words, it can be seen that the subject’s center of gravity pressure was biased to the left (red part of the heat map).

No significant differences were found in the area of center of pressure sway between age groups. However, time-based analysis in 10-min intervals revealed postural changes associated with fatigue. While normal seated posture exhibited symmetrical pressure across the ischial tuberosities, prolonged sitting led to individual-specific deviations including asymmetrical pressure, posterior pelvic tilt, increased micro-movements, and greater anterior–posterior sway (Figure 6).

### 3.3. HRV Indices

**Figure 7:**
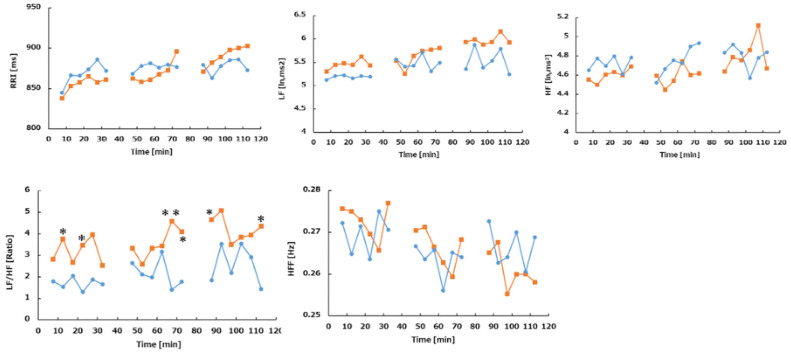
Comparison of HRV indices in phases 1–3. Orange indicates no oscillation (N = 9), blue indicates oscillation (N = 8) the result of the 40-min test. Of the 11 subjects, data was excluded from HRV analysis due to arrhythmia (with oscillation: 002, 004, 007, without oscillation 004, 007). HRV indices were calculated every 5 min using FFT. Data were excluded from analysis during PVT operation. In order to check whether there was a significant difference between oscillation and time change in each phase, comparison was performed using two-way ANOVA. * indicates *p* < 0.1 vs. oscillation+, ** indicates *p* < 0.05 vs. oscillation+. No significant difference in multiple comparisons. **Top left**: mean RR interval duration (RRI), **top middle**: low frequency component (LF, 0.04–0.15 Hz), **top right**: high-frequency component (HF, 0.15–0.40 Hz), **bottom left**: ratio of LF to HF (LF/HF), **bottom middle**: HF peak frequency (HFF): respiratory frequency.

**Figure 8:**
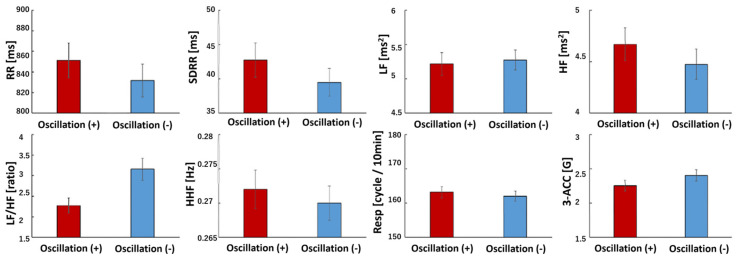
Biosignal data (analysis of Holter ECG, Oscillate + and Oscillate −). Oscillate (+): N = 99, Oscillate (−): N = 99, using paired *t*-test. The results of calculating the HRV index every 10 min are shown below. The red bar shows the average value of Oscillation (+), and the blue bar shows the average value of Oscillation (−). RRI is the RR interval of the electrocardiogram, SDRR is the standard deviation of the RR interval, LF is the low frequency component, HF is the high-frequency component, LF/HF is the ratio of LF to HF, HFF is the HF, Resp is the respiratory cycle, and 3-ACA is the three-axis acceleration (the value of the resultant acceleration). RRI (*p* = 0.004), SDRR (*p* = 0.015), LF (*p* = 0.426), HF (*p* = 0.02), LF/HF (*p* < 0.001), HFF (*p* = 0.413), Resp (*p* = 0.413), 3-ACA (*p* = 0.318). Compared with the case with vibration, the RRI, SDRR, and HF are significantly smaller, and the LF/HF is larger without vibration.

**Figure 9:**
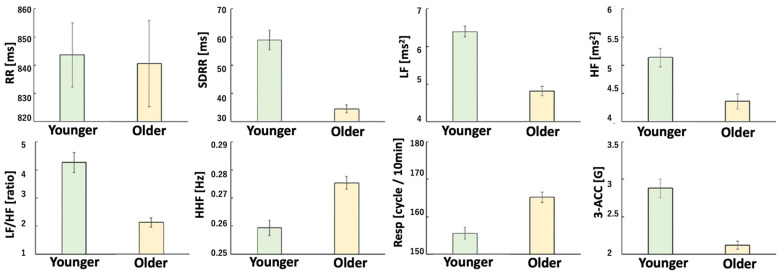
Biosignal data (analysis of Holter ECG, young vs. older characteristics). Younger: N = 54, Older: N = 144, independent Samples *t*-test. The green bar shows the average value of Younger, and the yellow bar shows the average value of Older. RRI (*p* = 0.875), SDRR (*p* < 0.001), LF (*p* < 0.001), HF (*p* < 0.001), LF/HF (*p* < 0.001), HFF (*p* < 0.001), Resp (*p* < 0.001), 3-ACA (*p* < 0.001). Regardless of the presence or absence of vibration, HRV disappeared in older people while watching the driving video. Sympathetic nerve activity was dominant in younger people. In addition, older people had a higher respiratory rate and younger people had a higher acceleration.

In the no-vibration condition, there was a trend toward increased LF power and a significant increase in LF/HF ratio in Phase 3 (*p* < 0.05), indicating heightened sympathetic activity. Compared to the vibration condition, the no-vibration condition showed significantly lower RRI, SDRR, and HF values and a higher LF/HF ratio (*p* = 0.004, *p* = 0.015, and *p* = 0.02, respectively). Regardless of vibration, older adults exhibited reduced HRV power, while younger adults showed dominant sympathetic activity. Additionally, older adults had higher respiratory rates, whereas younger adults displayed greater body acceleration (Figure 7, Figure 8 and Figure 9).

### 3.4. Body Acceleration (Physical Activity, PA)

**Figure 10:**
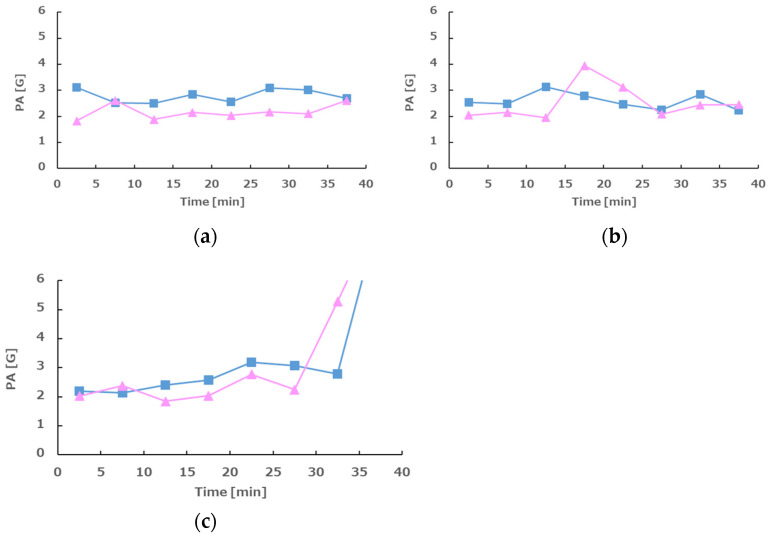
Physical activities for each phase. The graph shows the fluctuation of physical activity (PA). (**a**–**c**) show phases 1–3, respectively. The pink line shows the presence of vibration, and the blue line shows the absence of vibration. The vertical axis is G, and the horizontal axis is elapsed time (minutes). At the end of phase 3, the subject stands up after the measurement, so the acceleration increased.

Physical activity levels were low under both conditions (with and without vibration), but tended to be slightly higher without vibration (Figure 10).

### 3.5. Skin Temperature

**Figure 11:**
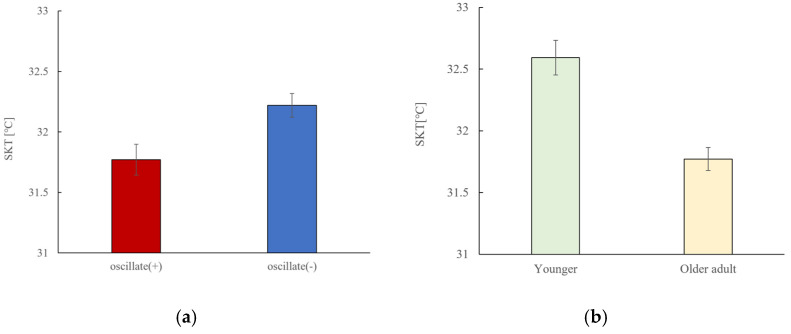
Skin temperature analysis (Oscillate + and Oscillate −, young vs. older characteristics). For (**a**), Oscillate (+): N = 99, Oscillate (−): N = 99, Paired *t*-test results. Average skin temperature was calculated every 10 min. Skin temperature was higher without vibration (*p* < 0.001). For (**b**), Younger: N = 54, Older: N = 144, Independent Samples *t*-test results. There are age differences in skin temperature (*p* < 0.001).

Skin surface temperature was significantly higher in the no-vibration condition compared to the vibration condition (*p* < 0.001) (Figure 11).

## 4. Discussion

This study explored the physiological and behavioral effects of low-frequency seat vibration during prolonged sitting in a simulated autonomous driving environment. The crossover design with 120-min sessions led to noticeable fatigue by the end of the experiment, particularly influencing postural control and autonomic regulation.

Postural stability and seat pressure dynamics; fatigue due to prolonged sitting appeared to reduce trunk and pelvic muscle strength, resulting in a shift in body weight to one side. This asymmetry was reflected in the seat pressure data as increased pressure on one ischial tuberosity and reduced pressure on the other. Additionally, posterior pelvic tilt and flattened lumbar curves (slouched posture) were observed in some participants. As fatigue progressed, participants unconsciously adjusted their posture to maintain comfort—either leaning forward or reclining—resulting in increased micro-movements and anterior–posterior sway. These changes likely contributed to increased mechanical load on the thighs and lower back. However, in the vibration condition, posture remained more stable throughout the phases, suggesting that rhythmic stimulation may contribute to postural maintenance.

PVT; no substantial differences in PVT performance were found between vibration and no-vibration conditions. Since PVT is a simple reaction time task, it may not be sensitive to subtle changes in arousal caused by external stimuli such as vibration. More cognitively demanding tasks might reveal clearer effects. The absence of a strong effect in the current study may be attributed to the vibration intensity, the monotony of the stimulus, characteristics of the PVT itself, inter-individual variability, or the influence of drowsiness. Future studies should consider combining vibration with more complex cognitive tasks and varying stimulation parameters. Although previous studies have investigated long-term driving [12,13,14,15,16,17], this study is novel in that it assessed alertness from the PVT. In addition, the possibility of increased false-positive responses in PVT performance should be considered. Such errors may arise from several sources: (1) fatigue, which reduces sustained attention and increases the likelihood of premature or incorrect responses; (2) momentary distraction, where participants briefly divert their attention from the task and respond inappropriately; (3) individual differences in vibration sensitivity, as some participants may experience heightened arousal or discomfort from vibration, leading to impulsive responses rather than true improvements in vigilance. These factors may interact in complex ways, complicating the interpretation of PVT outcomes. Future research should therefore not only evaluate mean reaction times but also analyze error patterns and response variability to better capture subtle cognitive effects of vibration.

Autonomic nervous system activity; HRV analysis revealed that the no-vibration condition led to significantly higher LF and LF/HF ratios in the final phase, with concurrent decreases in RRI, SDRR, and HF, indicating a shift toward sympathetic dominance. These findings suggest that prolonged static sitting without external stimulation may increase sympathetic nervous system activation due to accumulated fatigue, muscular tension, or discomfort. While drowsiness typically increases parasympathetic activity (HF), near-threshold drowsiness can trigger a compensatory rebound in sympathetic activity—a phenomenon known as the “micro-sleep defense mechanism.” This may explain the lack of major changes in PVT performance, as sympathetic activation could help preserve attention. Previous research on driving and heart rate has focused on evaluating drowsiness and stress [18,19,20,21,22,23,24], but this study adds a new perspective on fatigue and heart rate.

In contrast, the vibration condition maintained higher RRI, SDRR, and HF levels, suggesting enhanced parasympathetic tone and reduced stress. The consistent 2-Hz rhythmic stimulation may have served as a mild, continuous activator of the sympathetic system, preventing its overshoot and sustaining autonomic balance. This could help buffer the buildup of fatigue and stress during extended periods of sitting. A decrease in HF in the no-vibration condition may also reflect alterations in breathing patterns—possibly shallower or more irregular respiration—which should be further examined by analyzing respiratory cycles and their correlation with HRV.

Skin temperature; observed increase in skin temperature in the no-vibration condition may be attributed to heat accumulation due to restricted blood flow and reduced thermoregulatory responses such as sweating or vasodilation. Regarding the rise in skin temperature, in this experiment, the measurement time was in the afternoon, so the effects of circadian rhythms caused by this may also be considered. Moreover, vibration may influence peripheral circulation and thermoregulatory processes, leading to a transient decrease in skin temperature. One possible mechanism is that seat vibration enhances sympathetic nervous system activity, which in turn contributes to peripheral vasoconstriction and reduced cutaneous blood flow [25,26,27,28]. This vasoconstrictive response could temporarily lower skin temperature, reflecting an interaction between autonomic regulation [29,30,31] and mechanical stimulation. Further exploration of this hypothesis will require additional measurements such as peripheral blood flow and sweat rate. To date, various studies have been conducted on the biological load of car seats [22,23,24,32,33,34,35], vehicle vibration [17,36,37,38,39,40], and evaluation of drivers using a driving simulator [41], but this study provides a new perspective in that it analyzed multi-modal signals such as heart rate and seat pressure.

Limitations and future directions; this study has several limitations that should be acknowledged. First, the small sample size (n = 11) limits the generalizability of the findings, particularly with regard to age-based comparisons. While both young and older adults were included, the experiment was not originally designed to systematically evaluate age-related biological differences, but rather to assess physiological responses based on the presence or absence of vibration. In future studies, we aim to use more homogeneous age groups and larger sample sizes to more clearly evaluate such effects. Notably, the statistical power for detecting age-related effects was approximately 0.158 (preliminary), while the crossover design achieved a power of approximately 0.865, which supports the within-subject comparisons conducted in this study (the power analysis was conducted using GPower 3.1 with an alpha level of 0.05. Importantly, within-subject allows for the elimination of inter-individual differences that typically arise in between-subject designs. As a result, even with the same number of participants, the within-subject design yields higher statistical power). Second, in the vibration (Oscillation+) condition, seat pressure data were partially affected by mechanical interference from the vibration stimulus, which prevented accurate acquisition of continuous postural pressure measurements. As a result, the decline in postural stability over time was observed and confirmed primarily under the no-vibration (Oscillation−) condition. Although qualitative observations suggested more stable posture under vibration, direct and continuous quantitative evaluation of posture stability under the vibration condition was not possible. Therefore, interpretations regarding the benefits of vibration on postural control must be made cautiously, and we acknowledge the lack of direct evidence for improvement in posture stability under vibration. Third, we did not measure additional physiological markers such as respiratory rhythm, skin conductance, or peripheral blood flow, which could provide further insights into autonomic and thermoregulatory mechanisms. In particular, saliva cortisol testing—useful for monitoring fatigue accumulation—was excluded to avoid infectious disease risk, but will be incorporated in future experiments. Fourth, the vibration stimulus was limited to a fixed 2-Hz sinusoidal pattern, chosen to mimic the typical spontaneous breathing cycle (~4 s per cycle). However, as prior studies have noted, respiratory cycles vary widely across individuals and may not align with a fixed 2-Hz frequency [36]. Future research should investigate the effects of different vibration frequencies, amplitudes, and waveforms on autonomic regulation, postural control, and cognitive performance.

Although the primary hypothesis of this study was that vibration stimulation may help maintain wakefulness—using PVT as the main outcome—no statistically significant improvements in vigilance were observed. Contributing factors may include the following: (1) limited sample size; (2) an age-biased sample with predominance of older adults; (3) potential ceiling effects of task familiarity or vibration adaptation; (4) the 15-min washout period may have been insufficient for complete physiological recovery, particularly among older adults. In future crossover studies, it would be advisable to either extend the washout duration or incorporate physiological markers (e.g., heart rate variability, skin conductance) to objectively confirm recovery before transitioning between phases. This approach would help ensure that carryover effects do not confound the interpretation of experimental conditions. The current sample size did not allow for formal subgroup analyses; however, we emphasize the importance of such analyses in future work, particularly regarding potential age-related differences in physiological responses to vibration. Exploring these differences systematically may provide crucial insights into individualized countermeasures for fatigue in autonomous driving. A major limitation of this study is the absence of additional physiological markers that could help disentangle the effects of vibration on relaxation, stimulation, and stress. Future studies should incorporate autonomic and biochemical markers—such as salivary cortisol, skin conductance, and peripheral blood flow—to more precisely characterize underlying mechanisms. We plan to expand our experimental protocols to include these measures in order to distinguish between parasympathetic activation, arousal responses, and stress-related processes. Another important limitation is the lack of assessment of long-term effects. The present study was limited to short-term exposures and therefore could not capture the impact of accumulated fatigue, progressive adaptation, or potential habituation to vibration. To address this gap, future longitudinal studies with extended exposure periods are recommended to examine how rhythmic vibration influences vigilance, comfort, and musculoskeletal strain over time.

These results should therefore be regarded as preliminary and exploratory. The present study was designed primarily as a hypothesis-generating investigation, not a definitive test of efficacy. Moving forward, we plan to incorporate continuous respiratory monitoring to more accurately interpret changes in parasympathetic activity, and assess peripheral circulation to better explain the observed differences in skin temperature. In addition, we aim to combine vibration stimulation with cognitively demanding tasks to evaluate its effects on mental load and arousal. Ultimately, long-term field studies will be necessary to assess how rhythmic vibration affects comfort, fatigue, and musculoskeletal strain in autonomous driving and other prolonged sitting environments. To validate these findings under real-world driving conditions, it will also be essential to employ longer-term and ecologically valid protocols that incorporate multiple external stimuli and environmental challenges.

## 5. Conclusions

In this study, we investigated the physiological and behavioral effects of long-term sitting with or without the vibration stimulation of a car seat, and attempted to quantitatively evaluate it with a focus on psychomotor alertness, posture, autonomic nervous activity, body movement, and skin temperature. The vibration condition (Oscillation+) did not significantly improve performance on the psychomotor alertness task (PVT), but it was associated with an increase in the number of false starts, especially in older adults, suggesting that there are age-related differences in alertness responses to external stimuli. HRV analysis revealed that sympathetic nervous system activity increased over time in the no-vibration condition. In contrast, vibration stimulation is presumed to attenuate these stress responses, probably by maintaining a constant level of mild physiological stimulation. These findings suggest that mild, rhythmic vibration may help reduce physiological stress in long-term sitting and maintain postural stability. However, the interaction between vibration stimulation and autonomic function needs to be further elucidated, as the effects on cognitive performance, especially in simple reaction tasks, may differ by age and further investigation is needed.

## Figures and Tables

**Table 1:** Measurement Timeline and Data Collection.

Measure	Timing	Frequency
PVT	At the beginning and end of each phase	6 times total
Seat Pressure Mapping	At the beginning and end of each phase	6 times total
Skin Temperature	Continuously throughout each 120-min session	1-min averages
RRI/Body Accel.	Continuously throughout each 120-min session	Full-session recording

## Data Availability

The data presented in this study are available on request from the corresponding author. The data are not publicly available due to privacy or ethical restrictions but may be shared for research purposes upon reasonable request.

## Supplementary Materials

The following supporting information can be downloaded at §2: Pressure Distribution Sensor System (Takano Inc., Nagano, Japan, https://www.takano-net.co.jp/file/newtechnology/azwill.pdf accessed on 9 January 2025). §2: Holter ECG Device (Suzuken Co., Ltd., Nagoya, Japan—Cardy 303, 305 Pico. https://www.kenzmedico.co.jp/cardy305pico/ accessed on 9 January 2025). §2: Wrist-worn Biometric Sensor (TDK—Silmee™ W22, https://product.tdk.com/system/files/dam/doc/product/biosensor/biosensor/silmee_w22/ja/manual.pdf accessed on 9 January 2025).
